# Multimodality imaging in transcatheter aortic valve implantation: comparison between cardiovascular magnetic resonance, cardiac computed tomography, transesophageal and transthoracic echocardiography

**DOI:** 10.1186/1532-429X-14-S1-P98

**Published:** 2012-02-01

**Authors:** Andrew Jabbour, Tevfik F Ismail, Ali Vazir, Isabelle Roussin, Ankur Gulati, Francisco Alpendurada, Sameer Zaman, Oluwatosin Sotubo, Saman S Zaman, Ashwin Krishnamoorthy, Simon Davies, Sanjay K Prasad, Susanna Price, Michael B Rubens, Neil Moat, Raad H Mohiaddin

**Affiliations:** 1 NIHR Cardiovascular Biomedical Research Unit, Royal Brompton and Harefield NHS Foundation Trust, Imperial College London, London, UK; 2 Intensive Care Unit, Royal Brompton and Harefield NHS Foundation Trust, Imperial College London, London, UK; 3 Radiology, Royal Brompton and Harefield NHS Foundation Trust, Imperial College London, London, UK; 4 Cardiothoracic Surgery, Royal Brompton and Harefield NHS Foundation Trust, Imperial College London, London, UK

## Background

Patients often undergo several cardiac imaging investigations during assessment for (transcatheter aortic valve implantation) TAVI. Although data exists regarding the agreement between cardiovascular magnetic resonance (CMR), electrocardiograph-gated cardiac computed tomography (CCT) and transthoracic echocardiography (TTE) in TAVI patients, this study sought to determine the agreement and variability of transesophageal echocardiography (TEE) with these three modalities (CMR, CCT and TTE) in the assessment of aortic root morphology.

## Methods

Two hundred and two patients assessed by CMR, CCT and echocardiography for TAVI were studied. Agreement and variability between each imaging modality in the measurement of aortic annulus, sinus of Valsalva (SoV), sinotubular junction (STJ) and ascending aorta dimensions were assessed by Bland-Altman analysis. Intraobserver and interobserver variability was also assessed for CMR, CCT and echocardiography.

## Results

Of two hundred and two patients undergoing TAVI assessment with both CMR and TTE, one hundred and thirty three also underwent CCT, and fifty five TEE. Closest agreement was observed between CMR and CCT in dimensions of the aortic annulus (-0.4 (2.5) mm; -4.6 mm to 5.4 mm, (Bias (SD of Bias), 95% Limits of agreement)), SoV (-0.24 (2.25) mm; -4.7 mm to 4.2 mm), STJ (-0.8 (2.0) mm; -4.7 mm to 3.1 mm), and ascending aorta (-0.1 (2.2) mm, -4.4 mm to 4.2 mm).

Reasonably close agreement was also observed between CMR and TEE in dimensions of the aortic annulus (2.8 (2.4) mm; -1.9 mm to 7.4 mm, (Bias (SD of Bias), 95% Limits of agreement)), SOV (0.13 (2.8) mm; -5.4 mm to 5.7 mm), STJ (1.4 (2.0) mm; -2.6 mm to 5.4 mm), and ascending aorta (5.3 (3.3) mm, -1.3 mm to 11.8 mm).

Agreement between TTE-derived measures and CMR, CCT and TEE was less tight. CMR to TTE agreement in dimensions of the aortic annulus were (4.5 (3.3) mm; -1.9 mm to 11.0 mm), SOV (-0.45 (3.5) mm; -7.2 mm to 6.3 mm), STJ (-0.7 (3.9) mm; -8.4 mm to 7.0 mm) and ascending aorta (-1.8 (4.2) mm; -6.5 mm to 10.0 mm).

TTE to TEE agreement in dimensions of the aortic annulus were (-1.3 (3.5) mm; -8.2 mm to 5.5 mm), SOV (0.28 (3.4) mm; -6.3 mm to 6.9 mm), STJ (-2.2 (4.3) mm; -6.3 mm to 10.7 mm) and ascending aorta (-1.3 (4.6) mm; -7.7 mm to 10.3 mm).

Intraobserver and interobserver variability was lowest in CMR (p<0.01 for difference).

## Conclusions

In patients undergoing assessment for TAVI, closest agreement exists between CMR and CCT in the assessment of aortic root dimensions. Low intraobserver and interobserver variability was seen in both 3D-imaging modalities, although lowest for CMR. Lower agreement and higher variability was observed between TTE and CMR, CCT and TEE. Both TTE and TEE underestimated aortic annulus size when compared to both CMR and CCT.

## Funding

This project was supported by the NIHR Cardiovascular Biomedical Research Unit of Royal Brompton and Harefield NHS Foundation Trust, the British Heart Foundation, and CORDA. Dr. Jabbour was supported by a Postdoctoral Research Fellowship from the National Health and Medical Research Council of Australia, a Vincent Fairfax Family Foundation Research Fellowship from the Royal Australasian College of Physicians, the St. Vincent’s Clinic Foundation, and the Victor Chang Cardiac Research Institute.

